# Lactoferrin Dampens High-Fructose Corn Syrup-Induced Hepatic Manifestations of the Metabolic Syndrome in a Murine Model

**DOI:** 10.1371/journal.pone.0097341

**Published:** 2014-05-09

**Authors:** Yi-Chieh Li, Chang-Chi Hsieh

**Affiliations:** Department of Animal Science and Biotechnology, Tunghai University, Taichung, Taiwan; Institute of Medical Research A Lanari-IDIM, University of Buenos Aires-National Council of Scientific and Technological Research (CONICET), Argentina

## Abstract

Hepatic manifestations of the metabolic syndrome are related obesity, type 2 diabetes/insulin resistance and non-alcoholic fatty liver disease. Here we investigated how the anti-inflammatory properties of lactoferrin can protect against the onset of hepatic manifestations of the metabolic syndrome by using a murine model administered with high-fructose corn syrup. Our results show that a high-fructose diet stimulates intestinal bacterial overgrowth and increases intestinal permeability, leading to the introduction of endotoxin into blood circulation and liver. Immunohistochemical staining of Toll-like receptor-4 and thymic stromal lymphopoietin indicated that lactoferrin can modulate lipopolysaccharide-mediated inflammatory cascade. The important regulatory roles are played by adipokines including interleukin-1β, interleukin-6, tumor necrosis factor-α, monocyte chemotactic protein-1, and adiponectin, ultimately reducing hepatitis and decreasing serum alanine aminotransferase release. These beneficial effects of lactoferrin related to the downregulation of the lipopolysaccharide-induced inflammatory cascade in the liver. Furthermore, lactoferrin reduced serum and hepatic triglycerides to prevent lipid accumulation in the liver, and reduced lipid peroxidation, resulting in 4-hydroxynonenal accumulation. Lactoferrin reduced oral glucose tolerance test and homeostasis model assessment-insulin resistance. Lactoferrin administration thus significantly lowered liver weight, resulting from a decrease in the triglyceride and cholesterol synthesis that activates hepatic steatosis. Taken together, these results suggest that lactoferrin protected against high-fructose corn syrup induced hepatic manifestations of the metabolic syndrome.

## Introduction

Hepatic manifestations of the metabolic syndrome (HMMS) are associated with obesity, type 2 diabetes/insulin resistance, non-alcoholic fatty liver disease (NAFLD), and progression to non-alcoholic steatohepatitis (NASH) [1]. NAFLD is a common liver disease usually occurring in patients that do not habitually consume alcohol and manifesting as an excessive accumulation of triglycerides in the liver, leading to fat build-up and an increase in total liver weight of over 5%. The prevalence of fatty liver in obese and diabetic patients can reach 70–90% [2]. Fatty liver occurs either as simple steatosis (hepatic steatosis) or in combination with non-alcoholic liver inflammation. The resultant inflammation and liver cell damage may develop into liver fibrosis and progress to cirrhosis and even hepatocarcinoma. Histological changes in non-alcoholic and alcoholic fatty liver lesions mainly occur in the hepatic lobule, including fatty acid degeneration of liver cells, and fat accumulation of pathological features of the clinical symptom [3]. Sugar consumption has increased very rapidly in recent years in developed countries, and the prevalence of obesity and diabetes has parallely increased at an alarming rate. High-fructose corn syrup (HFCS) is used extensively in sugary drinks, especially in cola, soda, artificial juices, and other beverages. The hepatic metabolism of fructose begins with its phosphorylation by fructokinase. Fructose then directly enters the glycolytic pathway, bypassing the major control point by which glucose enters glycolysis. This unregulated carbon source provides glycerol-3-phosphate and acetyl-CoA for hepatic lipogenesis, increasing the hepatic pool of free fatty acids [4]–[6]. In addition, fructose neither suppresses ghrelin nor stimulates insulin or leptin to inhibit appetite [7]. According to a previous study, the prevalence of NAFLD in Taiwan ranges from 11–41%. Of the NAFLD patients, 6–13% were diagnosed with NASH. NAFLD has a severe impact on health that substantially increases when combined with obesity, diabetes, and the metabolic syndrome [8]. The presence of HFCS in beverages plays an important role in the progression of hepatic manifestations of the metabolic syndrome, including obesity, insulin resistance, NAFLD, and NASH.

In 1998, Day and James proposed the “double hit” hypothesis [9]. The first hit refers to the abnormal accumulation of lipids, especially triglycerides, in the liver. With the dysregulation of liver lipid homeostasis, free fatty acids (FFA) continue to be transported to the liver, resulting in a decreased capacity for β-oxidation of fats. In addition, most studies suggest that NASH is related to inflammation and insulin resistance. Further studies have shown that insulin resistance may lead to overexpression of the lipoprotein lipase (LPL) gene, thereby enabling continuous generation of free fatty acids in the liver [10]. Most patients may simply have a fatty liver with no associated inflammation. However, the “second hit” induces inflammatory responses, including abnormal inflammatory cytokine production and oxidative stress response [9]. Reactive oxygen species (ROS) activate redox-sensitive kinases, thereby activating IkappaB kinase beta (IKKβ), inducing nuclear factor-κB (NF-κB) activation, and further increasing the expression of TNF-α and production of cytokines by other inflammatory cells, leading to inflammation of the liver [11], [12]. Therefore, improvement of hepatic lipid metabolism and accumulation in “first-hit” and alleviating inflammation, insulin resistance and oxidative stress in “second-hit” have been shown the therapeutic potential in preventing the progression of HMMS.

Lactoferrin (Lf) is a single-chain glycoprotein consisting of 700 amino acid residues, with a molecular weight of 76–80 kD. It plays a variety of physiological roles, and mediates antibacterial, antiviral, and anti-inflammatory effects [13]. Lactoferrin exerts an antibacterial effect by binding iron ions, which reduces the iron-dependent growth of bacteria such as *E. coli* [13]. However, lactoferrin also acts as an iron donor to promote the growth of beneficial bacteria, such as *Lactobacillus* and *Bifidobacterium* [14], [15]. Inflammation or infection due to stimulation of phagocytes and release of cytokines further increases neutrophil infiltration. Lactoferrin is able to bind lipopolysaccharides (LPS), and is thereby able to reduce the LPS-driven inflammatory response [16]. It can also significantly reduce blood triglyceride and cholesterol levels and the peripheral fat mass in mice [17], [18]. In this study, we attempted to use lactoferrin to improve HFCS-induced HMMS including hepatic steatosis, insulin resistance, inflammation and oxidative stress in a murine model.

## Materials and Methods

### Reagents, ELISA Kits

Lactoferrin (90.5% pure with 16% iron saturation) was obtained from Westland Milk Products (Hokitika, New Zealand). Double-antibody sandwich ELISA kits to identify TNF-α, IL-1β, IL-4, IL-6, IL-13, IL-33, MCP-1, and TSLP (ELISA Ready-SET-Go; eBioscience, San Diego, CA, USA) were purchased. In addition, adiponectin (Mouse Adiponectin/Acrp30 DuoSet; R&D Systems Inc., Minneapolis, MN, USA), insulin (Ultrasensitive Mouse Insulin ELISA; Mercodia Inc., Winston, Salem, NC, USA), endotoxin (lipopolysaccharide) (ToxinSensor Chromogenic LAL Endotoxin Assay Kit; GenScript USA Inc., Piscataway, NJ, USA), and bovine lactoferrin (Bethyl Laboratories Inc., Montgomery, TX, USA) ELISA kits were purchased and according to the instruction to detect serum or hepatic homogenate.

### Animals and treatments

Fifty male C57BL/6JNarl mice (National Laboratory Animal Center, Taipei, Taiwan) were individually housed and maintained under environmentally controlled conditions (temperature 22–25°C, 12 h/12 h light/dark cycle, 45–60% humidity). The mice received a standard sterile rodent chow diet (Altromin Maintenance Irradiated Diet 1324 TFP, Altromin Spezialfutter GmbH & Co. KG, Lage, Germany) and distilled water *ad libitum*. The mice entered the experimental regimen at 6 weeks of age and were allowed 2 weeks to adapt. At 8 weeks of age, the mice were divided into 5 groups at the start of the experiments: naïve group: untreated; control group: HFCS-induced murine HMMS administered with distilled water; Lf treatment groups: HFCS-induced murine HMMS administered with lactoferrin at 50, 100, or 200 mg/kg/day. The body weight and food intake of each mouse was monitored on a weekly basis. After 8 weeks of treatment, all the mice were to fast for 8 h prior to measurement of blood glucose. Animals were anesthetized by isoflurane inhalation, and blood was collected *via* orbital sinus and centrifuged for 10 min at 3000 × *g* at 4°C to obtain serum, which was stored at −80°C until analysis. After blood collection, the mice were sacrificed using cervical dislocation and the livers removed, weighed, snap frozen in liquid nitrogen, and stored at −80°C. The experiment was performed in accordance with the Taiwan Animal Protection Act (2011), and the experimental protocol was approved by the Animal Welfare Committee of Tunghai University, Taichung, Taiwan (permit number: 101-6). All the surgeries were performed under isoflurane anesthesia, and all efforts were made to minimize suffering.

### Liver lipid extraction

Approximately 0.1 g of dissected liver was added to a 2-mL chloroform/methanol (2∶1, v/v) mixture and ground at room temperature for 1 h. The upper lipid layer was removed by centrifugation at 5,000 × *g* for 10 min. The lipid layer was further extracted by adding 0.2 volumes of 0.9% saline. After centrifugation at 5,000 × *g* for 5 min, the extract was dried under nitrogen at 55°C. The dry pellet was resuspended in 1 mL *tert*-butyl alcohol/Triton X-100/methanol (2∶1∶1, v/v) solution, collected, and stored at −20°C for subsequent analysis.

### Determination of alanine aminotransferase, cholesterol, and triglyceride levels

A 50-µL sample of serum was obtained from blood by centrifugation at 1700 × *g* for 10 min at 4°C. Serum alanine aminotransferase (ALT), cholesterol, and triglyceride levels were measured using clinical kits (Roche Diagnostics, Mannheim, Germany) and a spectrophotometric system (Cobas Mira; Roche, Rotkreuz, Switzerland). Liver lipid extracts were measured using the same method.

### Oral glucose tolerance test

At the end of dietary intervention, an oral glucose tolerance test (OGTT) was performed after an overnight fast. Each mouse was intragastrically administered with a dose of 2 g/kg body weight of D-glucose. Blood samples (5 µL) were obtained from the caudal vein to measure blood glucose levels at 0, 30, 60, 90, and 120 min using a CareSens II Blood Glucose Monitoring System and Test Strips (i-SENS Inc., Seoul, Korea).

### Estimation of cytokines, insulin, endotoxin, and bovine lactoferrin in homogenized liver and serum

To study the effect of lactoferrin on liver and serum cytokines in HFCS-induced NASH mice, double-antibody sandwich ELISAs (for TNF-α, IL-1β, IL-4, IL-6, IL-13, IL-33, MCP-1, TSLP, adiponectin, insulin, endotoxin, or bovine lactoferrin) were performed according to the manufacturers'instructions. The frozen liver was thawed, and approximately 0.1 g of liver tissue removed and homogenized in 1 mL of buffer (1.15% KCl/Tris-EDTA pH 8.9 acetic acid 3∶2∶1.5 v/v), followed by centrifugation at 4500 × *g* for 10 min at 4°C. The supernatant was collected and stored at −80°C for subsequent analysis. Capture antibodies were added to 96-well plates and incubated overnight at 4°C. Each sample well was washed three times, blocked for 60 min at room temperature, and washed three more times. Standards and samples at dilutions of 1∶10–1∶100 were then added to the wells. After a 2-h incubation at room temperature, the wells were washed five times. After applying the detection antibodies and incubating for 1–2 h, the wells were washed seven times to remove non-specific binding. Substrate solution (TMB) was added to each well, and following incubation in the dark at room temperature for 15 min, stop solution was added to terminate enzyme activity. Absorbance was measured at 450 nm, with 570 nm as the reference wavelength, using an ELISA reader (Multiskan Spectrum, Thermo Electron Corporation, San Jose, CA, USA).

### Insulin Sensitivity Test

Homeostasis model assessment-insulin resistance (HOMA-IR) was determined using the steady-state blood glucose and insulin concentrations in a feedback interaction loop. HOMA-IR was calculated using the relationship between the blood glucose and insulin levels, according to the following formula:

HOMA-IR  =  Insulin (µU/L) × Blood glucose (mM)/22.5 [19]

### Pathological examination

After formalin fixation, tissue samples were sliced (5-µm sections), embedded in the standard manner, and stained with hematoxylin & eosin (HE). Fatty liver was graded according to the method of Kleiner *et al*., 2005 [20], and using a low- to medium-power evaluation of standard HE-stained liver parenchyma to measure percentage parenchymal involvement by steatosis, graded as follows: grade 0, <5% steatosis; grade 1, 5–33% steatosis; grade 2, 33–66% steatosis; grade 3, >66% steatosis. To avoid sampling errors, all the biopsies were performed on the same lobe, and the semi-quantitative grades were assigned without knowledge of the sample treatment.

Frozen liver sections (10-µm) were embedded in FSC 22 Frozen Section Media (Surgipath, Leica Biosystems, Richmond Inc., IL, USA) and stained with Oil Red O to visualize hepatic lipids. Images of the red-stained lipid areas were enhanced with Adobe Photoshop CS6, and the size and number of lipid droplets were analyzed using ImagePro Plus 5.0, as previously described [21], [22]. A minimum of 5 separate microscopic fields of view were used to measure the lipid droplet area of each section, at a magnification of 400×.

### Immunohistochemical staining

The expression and localization of 4-hydroxynonenal (4-HNE), Toll-like receptor-4 (TLR-4), and thymic stromal lymphopoietin (TSLP) in the liver were determined by immunohistochemical staining, as described previously [23]. For primary staining of 4-HNE, TLR-4, and TSLP, deparaffinized tissue sections were incubated with polyclonal anti-4-HNE (MyBioSource, Inc., San Diego, CA, USA), anti-TLR-4, or anti-TSLP (Biosis, Inc., Woburn, MA, USA) antibodies. A secondary antibody (HRP-conjugated mouse anti-goat immunoglobulin G fragment antibody) was added, and the specific staining was visualized using an immunodetection kit and a 3,3-diaminobenzidine chromogen (Novolink Max Polymer Detection System, Leica Biosystems Newcastle Ltd, Newcastle Upon Tyne, United Kingdom).

### Statistical analysis

Results were expressed as mean ± SD. One-way ANOVA was used for multiple group comparisons, and Duncan's test was used for post hoc examination. Data for steatosis histopathological scores were presented as means and were analyzed using the non-parametric test, followed by a Mann–Whitney *U*-test to compare group differences. Differences with *P*<0.05 were considered significant.

## Results

### Body, liver, and spleen weight

The basic physiological data for 30% (v/v) HFCS-induced HMMS are shown in Table 1. After eight weeks, the control group (HFCS without lactoferrin) showed significant increases in body, liver, and spleen weight, as well as a body weight gain (*P*<0.05). The lactoferrin treatment groups (50, 100, and 200 mg/kg) showed significantly lower body, liver, and spleen weights, as well as body weight gain (*P*<0.05). The control group exhibited significant swelling, and further increase in portal hypertension induced spleen swelling.

**Table 1:** Effects of lactoferrin on body, liver, and spleen weights, and weight gain in HFCS-induced murine HMMS.

	Naïve	HFCS-induced murine HMMS			
		Control, DW	Lf, 50 mg/kg	Lf, 100 mg/kg	Lf, 200 mg/kg
**Pre-treatment body weight (Pre-BW), g**	23.24±1.12	24.63±1.90	24.35±1.71	24.10±1.60	24.85±1.08
**Post-treatment BW (Post-BW), g**	24.90±1.14^ a^	33.23±2.44^ c^	27.71±2.29^ b^	28.14±2.00^ b^	27.63±1.96^ b^
**Weight gain, g**	1.66±0.35^ a^	8.60±1.75^ d^	3.36±0.84^ bc^	4.04±0.87^ c^	2.78±1.34^ b^
**Liver weight (LW), g**	0.94±0.08^ a^	1.37±0.12^ b^	1.08±0.06^ a^	0.96±0.31^ a^	1.00±0.16^ a^
**Spleen weight (SW), mg**	62.83±5.47^ a^	94.01±8.84^ d^	77.21±9.76^ bc^	80.34±11.64^ c^	70.75±10.42^ ab^
**LW/Post-BW, %**	3.80±0.30^ ab^	4.12±0.17^ b^	3.91±0.36^ ab^	3.39±1.10^ a^	3.62±0.41^ ab^

Following 8 weeks of lactoferrin administration (0, 50, 100, and 200 mg/kg), body, liver, and spleen weights, and weight gain were significantly higher in the control group than in the naïve group (*P*<0.05). Body, liver, and spleen weights, and weight gain were significantly lower in the lactoferrin-treated groups than in the control group (*P*<0.05). Data are presented as mean ± SD (n = 10), and were analyzed using one-way ANOVAs and Duncan's multiple range test. ^a–d^: Different letters in the same row indicate a significant difference (*P*<0.05).

### Fatty liver and hepatic damage

**Figure 1:**
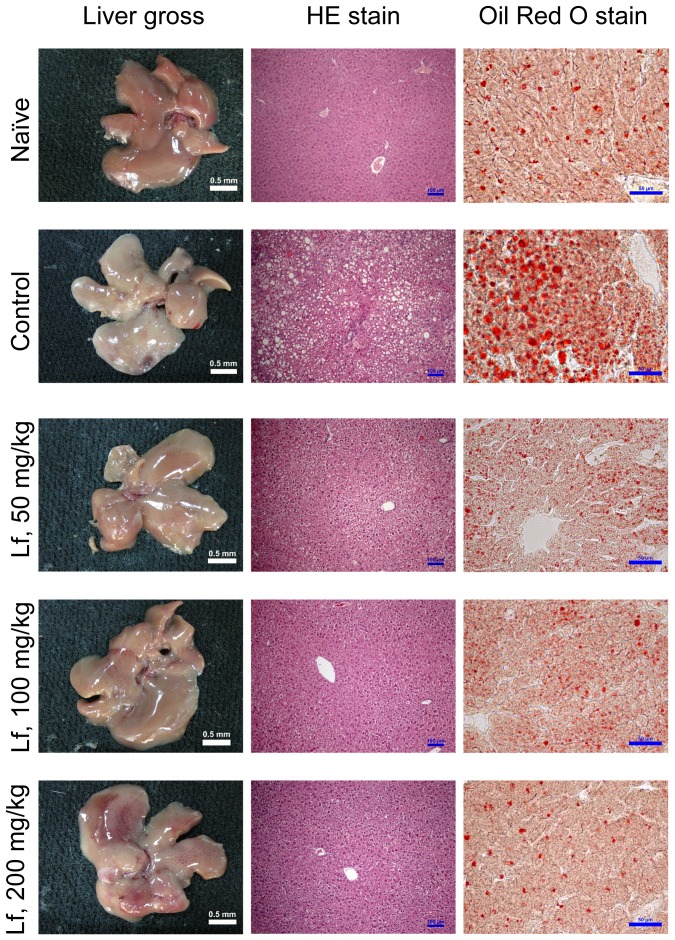
Gross and microscopic evaluation of the effect of lactoferrin on lipid accumulation by the mouse liver after HFCS challenge. Liver sections (5 µm) were prepared using HE and Oil Red O stains to determine lipid accumulation. Lipid accumulation around the vein was minimal in normal mice (naïve group). Lipid accumulation was marked in HMMS mice (control group). Treatment with lactoferrin at 50, 100, and 200 mg/kg markedly suppressed lipid accumulation. The scale bar represents 0.5 mm in liver gross, 100 µm in HE-stained sections, and 50 µm in Oil Red O stained sections.

**Figure 2:**
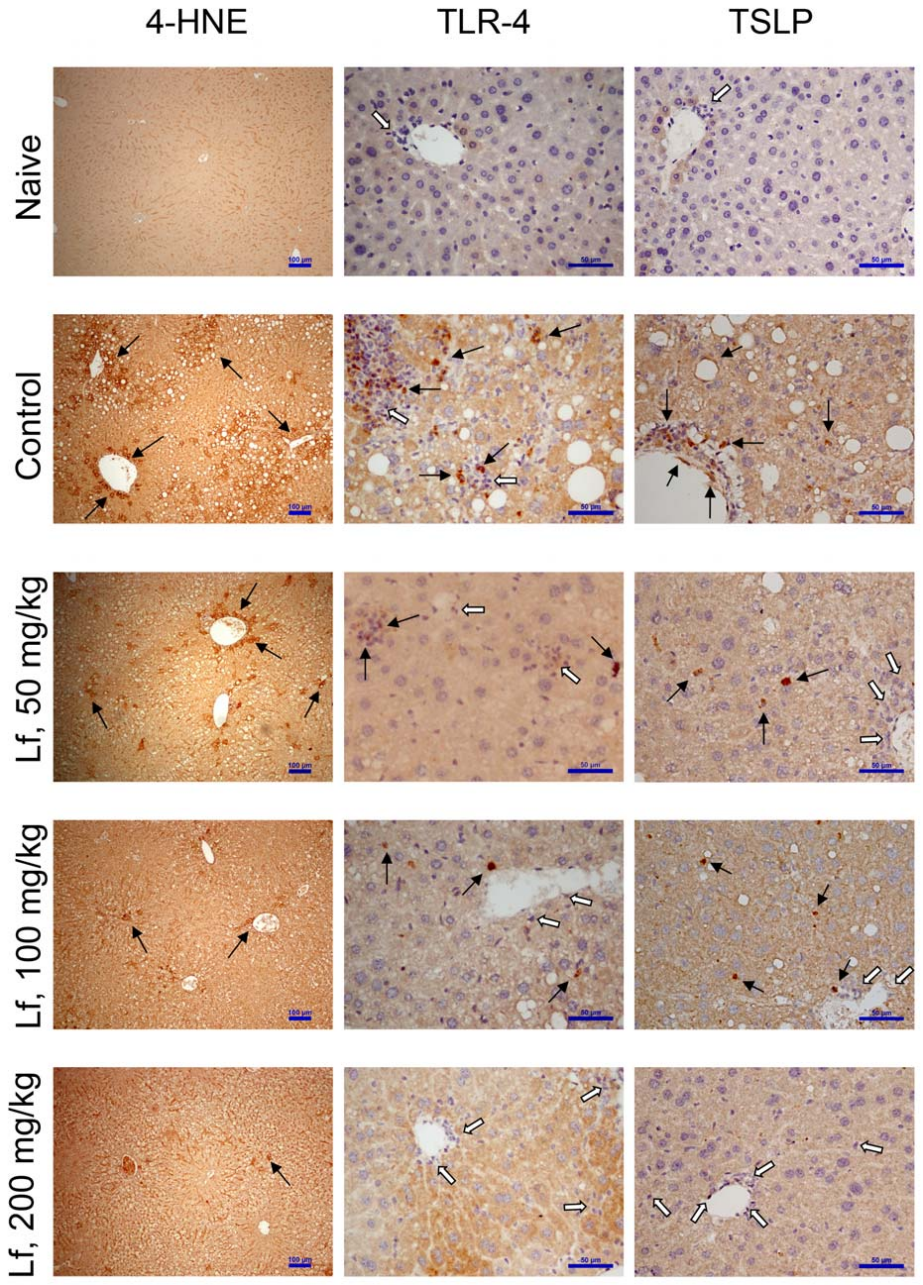
Treatment with lactoferrin improved the histology of livers of HFCS-induced HMMS mice. Immunohistochemical staining with 4-HNE, TLR-4, and TSLP show lipid peroxidation (4-HNE) and associated inflammatory markers (TLR-4 and TSLP). The control group showed lipid vascular accumulation and stained positive for 4-HNE, TLR-4, and TSLP, compared to the naïve group (indicated by arrows). Open arrows indicate negative staining for TLR-4 and TSLP, which indicate without infiltration of inflammatory cells. Treatment with lactoferrin at 50, 100, and 200 mg/kg markedly reduced 4-HNE, TLR-4, and TSLP expression. Scale bars represent 100 µm (4-HNE) and 50 µm (TLR-4 and TSLP).

After eight weeks of lactoferrin administration (50, 100, and 200 mg/kg) to mice with HFCS-induced HMMS, the mice were sacrificed, and their livers subjected to gross and microscopic examination. Mice from the control group (no lactoferrin) had fatty livers, while lipid accumulation was markedly reduced in the groups administered with lactoferrin (Figure 1). HE staining of paraffinized sections showed oil droplet vacuoles in the control group that were reduced in the lactoferrin treatment groups. A similar effect was shown in the frozen sections stained with Oil Red O (Figure 1). The control group's histopathological steatosis score was significantly higher than naïve group (Table 2; *P*<0.001). The lactoferrin treatment groups (50, 100, and 200 mg/kg) had significantly lower histopathological steatosis scores than the control group (Table 2; *P*<0.01). In the measurement of Oil Red O stain, lipid droplet area (%) and numbers were significantly lower in the lactoferrin-treated groups (Table 3; *P*<0.05). The hepatic homogenate was analyzed to measure fat accumulation. Hepatic triglyceride (hTG) and serum triglyceride (sTG) levels were significantly reduced in the lactoferrin treatment groups compared to the control group (Table 4, 5; *P*<0.05). In addition, serum concentrations of total cholesterol (sCHOL) were significantly increased in the HFCS-induced NASH control group (Table 4; *P*<0.05). Administration of lactoferrin (50, 100, and 200 mg/kg) significantly reduced sCHOL levels compared to the control group (Table 4; *P*<0.05). Severe lipid accumulation results in lipid peroxidation and inflammation. 4-HNE is an important indicator of lipid peroxidation, and immunohistochemical staining showed that 4-HNE staining in the liver tissue of the control group was significantly higher, but significantly reduced by lactoferrin treatment (Figure 2). In the control group, sALT increased significantly (Table 4; *P*<0.05), but reduced by lactoferrin administration (Table 4; *P*<0.05). These data indicate that lactoferrin was significantly reduced oil droplet accumulation and liver damage.

**Table 2:** Effect of lactoferrin on hepatic steatosis scores.

Group	Numbers of mice with different steatosis scores				Mean	*P*
	−	+	++	+++		
Naïve	10	0	0	0	0	
HFCS-induced HMMS						
Control	0	0	2	8	2.8^###^	0.000
Lf, 50 mg/kg	0	2	6	2	2.0**	0.007
Lf, 100 mg/kg	0	3	7	0	1.7***	0.000
Lf, 200 mg/kg	0	6	4	0	1.4***	0.000

Following eight weeks of lactoferrin administration (0, 50, 100, and 200 mg/kg), the control group had a significantly higher histopathological steatosis score than the naïve group (^###^ *P*<0.001). The lactoferrin-treated groups had significantly lower steatosis scores than the control group (**P<0.01, ***P<0.001). Data are presented as means and were analyzed with non-parametric statistics followed by the Mann–Whitney *U*-test to compare group differences. Grade designations of the histological findings are: (−, 0) normal, (+, 1) slight, (++, 2) moderate, and (+++, 3) severe steatosis. Each value represents the number of animals that showed a change in grade during the experimental period.

**Table 3:** Effect of lactoferrin on liver lipid droplets in Oil Red O stain.

	Naïve	HFCS-induced murine HMMS			
		Control	Lf, 50 mg/kg	Lf, 100 mg/kg	Lf, 200 mg/kg
Area, %	3.0±0.4^ a^	18.8±5.3^ c^	6.1±2.6^ b^	4.7±1.9^ ab^	4.2±1.3^ ab^
Number	29.6±2.7^ a^	146.6±12.4^ b^	36.3±6.1^ a^	30.8±4.7^ a^	30.1±4.0^ a^

Following eight weeks of lactoferrin administration (0, 50, 100, and 200 mg/kg), the area (%) and number of lipid droplets were significantly higher in the control group than the naïve group (*P*<0.05). The area (%) and number of lipid droplets were significantly lower in the lactoferrin-treated groups than in the control group (*P*<0.05). Data are presented as the mean ± SD (n = 10), and were analyzed using one-way ANOVAs and Duncan's multiple range test. ^a–c^: Different letters in the same row indicate a significant difference (*P*<0.05).

**Table 4:** Effects of lactoferrin on serum mediators in HFCS-induced murine HMMS.

	Naïve	HFCS-induced murine HMMS			
		Control	Lf, 50 mg/kg	Lf, 100 mg/kg	Lf, 200 mg/kg
sALT, U/L	16.50±6.26^ a^	75.50±39.89^ c^	40.50±7.98^ b^	31.00±21.32^ ab^	24.00±10.49^ ab^
sTG, mg/dL	26.90±13.26^ a^	61.15±7.13^ b^	35.40±14.13^ a^	32.65±15.11^ a^	34.85±9.66^ a^
sCHOL, mg/dL	57.50±8.90^ a^	94.00±15.06^ c^	75.00±19.86^ b^	75.50±21.79^ b^	74.50±16.91^ b^
sLPS, EU/mL	0.16±0.03^ a^	0.62±0.01^ d^	0.46±0.02^ c^	0.42±0.02^ b^	0.40±0.01^ b^
sbLf, µg/mL	1.99±0.36^ a^	2.24±0.40^ a^	4.38±0.38^ b^	7.70±0.92^ c^	12.04±2.64^ d^

Following eight weeks of lactoferrin administration (0, 50, 100, and 200 mg/kg), levels of serum LPS (sLPS), serum ALT (sALT), serum triglyceride (sTG), serum cholesterol (sCHOL), and serum bovine lactoferrin (sbLf) were significantly higher in the control group than in the naïve group (*P*<0.05). sLPS, sALT, sTG, and sCHOL were significantly lower in the lactoferrin-treated groups than in the control group (*P*<0.05). sbLf was significantly higher in the lactoferrin-treated groups than in the control group, in a dose-dependent manner. Data are presented as the mean ± SD (n = 10), and were analyzed using one-way ANOVAs and Duncan's multiple range test. ^a–d^: Different letters in the same row indicate a significant difference (*P*<0.05).

**Table 5:** Effects of lactoferrin on hepatic mediators in HFCS-induced murine HMMS.

	Naïve	HFCS-induced murine HMMS			
		Control	Lf, 50 mg/kg	Lf, 100 mg/kg	Lf, 200 mg/kg
hTG, mg/g	9.9±4.4^ a^	33.4±9.0^ b^	10.4±3.5^ a^	10.8±6.1^ a^	13.9±5.3^ a^
hIL-1β, ng/g	53.3±30.5^ a^	132.4±63.9^ b^	54.7±16.0^ a^	57.8±24.3^ a^	40.6±19.0^ a^
hIL-6, ng/g	16.8±4.6^ a^	23.8±6.2^ b^	20.5±8.6^ ab^	15.8±4.6^ a^	18.8±3.6^ ab^
hTNF-α, ng/g	97.9±66.4^ a^	175.2±63.9^ b^	109.8±41.7^ a^	93.9±42.9^ a^	121.4±52.7^ a^
hMCP-1, ng/g	115.0±64.8^ a^	223.7±55.2^ b^	133.9±26.5^ a^	128.0±65.8^ a^	142.3±44.3^ a^
hIL-4, ng/g	50.9±26.9^ a^	117.5±41.1^ b^	76.8±16.7^ a^	66.9±31.0^ a^	69.1±23.2^ a^
hIL-13, µg/g	14.6±6.1^ a^	24.5±7.9^ b^	16.5±2.7^ a^	15.7±5.7^ a^	15.3±5.3^ a^
hIL-33, ng/g	276.3±124.1^ a^	431.8±192.8^ b^	266.6±73.0^ a^	233.2±96.7^ a^	261.7±100.8^ a^
hTSLP, ng/g	81.6±17.3^ a^	133.7±36.6^ b^	100.8±40.6^ a^	83.5±22.3^ a^	93.0±39.9^ a^
hAdn, mg/g	5.8±1.1^ c^	3.7±1.3^ a^	4.3±1.2^ ab^	4.9±0.7^ bc^	5.7±0.9^ c^
hLPS, EU/g	2.8±0.4^ a^	4.3±0.8^ b^	3.1±0.5^ a^	3.0±0. 5^ a^	2.7±0.2^ a^
hbLf, µg/g	7.3±1.4^ a^	8.9±1.3^ a^	14.2±1.7^ b^	17.2±9.2^ b^	22.8±5.1^ c^

Following eight weeks of lactoferrin administration (0, 50, 100, and 200 mg/kg), levels of hepatic LPS (hLPS), hTG, hIL-1β, hIL-6, hTNF-α, hMCP-1, hIL-4, hIL-13, hIL-33, and hTSLP were significantly higher in the control group than in the naïve group (*P*<0.05). Hepatic adiponectin (hAdn) was significantly lower in the control group than in the naïve group (*P*<0.05). There was no significant difference in hepatic bovine lactoferrin (hbLf) between the control and naïve groups. hLPS, hTG, hIL-1β, hTNF-α, hMCP-1, hIL-4, hIL-13, hIL-33, and hTSLP were significantly lower in the lactoferrin treatment groups (50, 100, and 200 mg/kg) than the control group (*P*<0.05). hIL-6 was significantly lower than the control only in the lactoferrin group receiving 100 mg/kg. hAdn was significantly higher than the control group in the 100 and 200 mg/kg lactoferrin groups (*P*<0.05). hbLf was significantly higher in the lactoferrin groups than the control group, in a dose-dependent manner. Data are presented as the mean ± SD (n = 10), and were analyzed with one-way ANOVAs and Duncan's multiple range test. ^a–d^: Different letters in the same row indicate a significant difference (*P*<0.05).

### Inflammatory adipokines in liver damage

Inflammatory adipokines including cytokines, chemokines and acute phase proteins that are involved in adipocyte development. The liver contains a variety of cell types, which play roles in defense and detoxification, or which help form the structural matrix. Hepatic IL-1β (hIL-1β), hTNF-α, and hMCP-1 are primarily produced by Kupffer cells and neutrophils. In our present results, these inflammatory adipokines were significantly increased in the control group compared to the naïve group (Table 5; *P*<0.05), but not in the lactoferrin-treated groups (Table 5; *P*<0.05). Compared to the naïve group, levels of hIL-4 and hIL-13 were significantly increased in the control group (Table 5; *P*<0.05), but not in the lactoferrin-treated groups (Table 5; *P*<0.05). Epithelial cell-derived cytokines, including IL-33 and TSLP, modulate inflammation; hIL-33 and hTSLP levels were significantly increased in the control group compared to the naïve group (Table 5; *P*<0.05), but not in the lactoferrin-treated groups (Table 5; *P*<0.05). Finally, adiponectin (Adn) antagonizes adipocyte maturation, and the hAdn level was significantly reduced in the control group (Table 5; *P*<0.05) but not in the 100 and 200 mg/kg lactoferrin-treated groups (Table 5; *P*<0.05). TLR-4 and TSLP had been detected in inflammatory tissue by immunohistochemical staining and were significantly increased in the control group relative to the naïve group, but not in the lactoferrin-treated groups (Figure 2).

### Endotoxin and bovine lactoferrin (bLf) levels in liver and blood

Endotoxin levels were significantly increased in the liver and blood of mice with HFCS-induced HMMS (Table 4, 5; *P*<0.05). After administration of various doses of bLf (50, 100, and 200 mg/kg), endotoxin levels in murine liver and blood significantly decreased (*P*<0.05). Lactoferrin therefore plays a role in scavenging endotoxins, such that the liver and blood levels in HFCS-induced NASH mice were not significantly different from that in naïve control mice. Oral administration of bLf (50, 100, and 200 mg/kg) resulted in significant increases in bLf levels in liver homogenates and serum (Table 4, 5; *P*<0.05).

### HMMS-related insulin resistance

**Figure 3:**
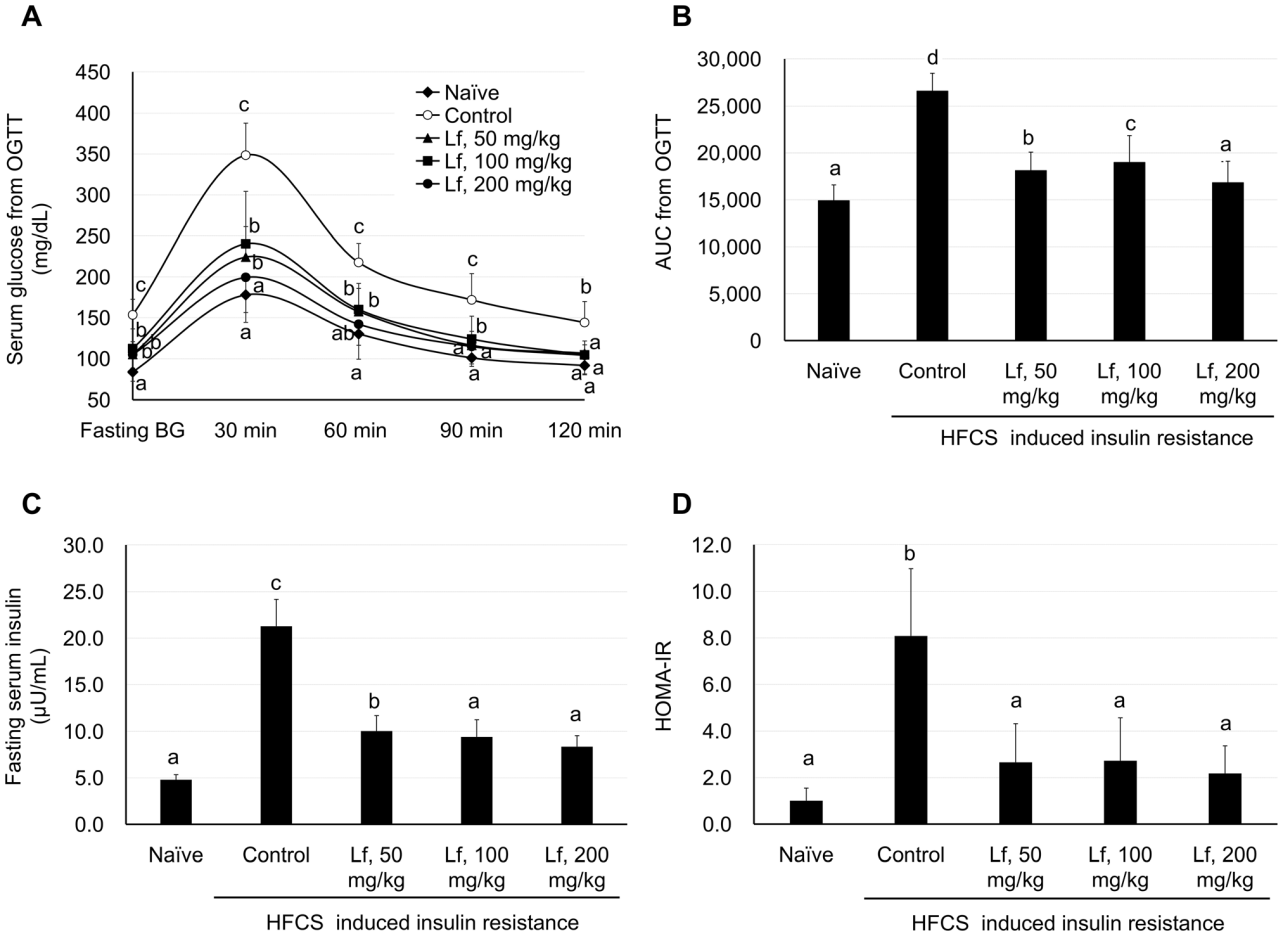
Insulin resistance and OGTT determination in HFCS-induced insulin resistance in the murine model. (A) Blood glucose determined by OGTT after oral administration of glucose (2%). (B) Area under curve (AUC) was calculated for the OGTT. The control group showed a significant increase compared to the naïve group, whereas lactoferrin at 50, 100, and 200 mg/kg markedly reduced the AUC. (C) Fasting serum insulin was determined by ELISA. Insulin levels were significantly increased in the control group compared to the naïve group, and lactoferrin at 50, 100, and 200 mg/kg markedly reduced blood insulin. (D) HOMA-IR insulin resistance calculated from fasting serum glucose and insulin levels. Lactoferrin at 50, 100, and 200 mg/kg markedly reduced blood insulin. Data are presented as mean ± SD (n = 10) and were analyzed using one-way ANOVAs and Duncan's multiple range test. ^a–d^: Different letters indicate significant differences between groups (*P*<0.05).

The hepatic manifestations of the metabolic syndrome are fatty liver and/or hepatic steatosis linked to insulin resistance. Type 2 diabetes is also a chronic inflammatory condition characterized by elevation of concentrations of ROS and endotoxins. As shown in Figure 3A, measurements of serum glucose by OGTT demonstrated that compared to the naïve group, the control group had higher blood glucose concentrations after fasting and 30, 60, 90, and 120 min after oral glucose administration (Figure 3A; *P*<0.05). The lactoferrin-treated groups showed significantly lower blood glucose levels at all times compared to the control group. Area under curve (AUC) measurements from the OGTT indicated that glucose levels were significantly higher in the control group than in the naïve group, and that glucose levels were significantly lower in the lactoferrin-treated groups than in the control group (Figure 3B; *P*<0.05). Fasting insulin levels were significantly reduced in the lactoferrin-treated groups, in a dose-dependent manner (Figure 3C; *P*<0.05). The homeostatic model assessment (HOMA) is a method used to determine insulin resistance (HOMA-IR). Our data indicate that the lactoferrin-administered groups had significantly reduced HOMA-IR (Figure 3D; *P*<0.05), suggesting that lactoferrin can lower insulin resistance.

## Discussion

In the western diet, long-term consumption of beverages containing HFCS contributes to the development HMMS, including obesity, insulin resistance, hypertriglyceridemia, NAFLD, and NASH, all of which are associated with an inflammatory state. Previous studies have indicated that administration of 15–54% aqueous solutions of HFCS can elevate fasting blood glucose concentrations and increase insulin resistance in murine models [24], [25]. Inflammatory status indicates the progression of obesity, insulin resistance, NAFLD, and NASH [26]. In this study, gross examination of the livers of HFCS-administered mice showed lipid accumulation and a 45% increase in liver weight (1.37 g vs. 0.94 g; Table 1). Microscopic examination of liver sections revealed steatosis after HFCS administration, which was significantly reduced in a dose-dependent manner by lactoferrin treatment (Figure 1). After 8 weeks of HFCS induction, severe steatosis (>80%) was observed. However, there were no severe steatosis could be detected in mice administered with the higher doses of lactoferrin (100 and 200 mg/kg; Table 2). Oil Red O staining also showed that lactoferrin administration could significantly reduce lipid droplet accumulation. Serum and hepatic triglyceride (sTG and hTG) levels significantly increased after HFCS induction; lactoferrin treatment lowered sTG and hTG levels to the normal range (naïve group; Tables 4, 5). High serum cholesterol, another HFCS-induced HMMS, could also be reduced by lactoferrin administration (Table 5). Previous studies using the murine model have shown that lactoferrin can also significantly reduce triglycerides and cholesterol in the plasma and fat tissue mass [18], [27]. As mentioned earlier, the first hit described by the “double hit” hypothesis is triglyceride accumulation in the liver [9]. Ishimoto *et al*. used fructokinase (Ketohexokinase-A and -C) knockout mice (KHK-A/C KO) to demonstrate that fructokinase plays a central role in the first hit, via the development of steatohepatitis resulting from a high-fat and high-sucrose (Western) diet [28]. In the present study, sTGs and hTGs were reduced to normal levels in the lactoferrin-treated groups (Table 4, 5). Histopathological analysis of Oil Red O staining also indicated that administration of lactoferrin significantly reduced the area and number of lipid droplets (Table 3, Figure 1). These results indicated that lactoferrin or lactoferrin hydrolysate could alter the transformation of fructose to fructose-1-phosphate *via* antagonism of fructokinase activity. Johnson *et al*. developed a method for fructokinase inhibition screening (U.S. Patent application: US20130195886A1) that involves the provision of fructokinase-C and -A inhibitors, including (z)-3-(methylthio)-1- phenyl-N′-(((4-trifluoromethoxy)phenyl)carbamyoyl) oxy)-1H-pyrazole-4-carboximidamide, 5-amino-3-(methylthio)-1-phenyl-1H-pyrazole -4-carbonitrile, and 2-(3-(methylthio)-1-phenyl-1H-pyranol-4-yl)-4-phenylthiazole, which are phenyl-ring enriched. The sequence of lactoferrin is rich in aromatic-ring peptides (phenylalanine, tyrosine, and tryptophan), which might be the functional peptides in the digestive hydrolysate. Fernández-Musoles *et al*. predicted that the functional oligo-peptides (LIWKL and RPYL) of lactoferrin have antihypertensive activity, and subsequently demonstrated that the antihypertensive activity of RPYL (200 µM) is equal to that of valsartan (0.1 µM) [29]. We predict that oligopeptides with aromatic rings, including PEWF, PYFGY, PYKLRP, PQTHYY, FQLFGSPP, and VVWCAVGP, would be showed fructokinase-antagonistic activity; we are going to investigate these oligopeptides in the further studies. Elevated lipid peroxidation can be detected by measuring 4-HNE, which along with malondialdehyde, arises from increases in the oxidative stress chain reaction. HCFS increased the 4-HNE accumulation observed in tissue sections, and its production was significantly reduced by lactoferrin treatment (Figure 2). There were no previous study indicate that lactoferrin reduces 4-HNE formation in tissues under hyperoxidative stress conditions. However, the role of lactoferrin play a part of defense mechanism against lipid peroxidation may explain why sputum lactoferrin levels are higher in smokers than in non-smokers [30]. Our study presents the novel observation that lactoferrin can reduce the 4-HNE protein adducts produced by the oxidative stress chain reaction.

Hepatic manifestations of the metabolic syndrome caused by HFCS administration also include insulin resistance. Fasting insulin, blood glucose, and HOMA-IR were significantly increased in serum after HFCS induction for 7 weeks (Figure 3A, 3C, 3D). Fasting insulin and blood glucose did not fall to the levels observed in the naïve group after treatment with lactoferrin (Figure 3A, 3C). But HOMA-IR of lactoferrin-treated groups were reduced to the level as in the naïve group (*P*>0.05, Figure 3D). Examination of the OGTT, an indicator of insulin insensitivity, showed the lactoferrin groups had significantly lower glucose levels than the control group at 30, 60, 90, and 120 min. At 120 min after oral administration of 2% glucose to fasting mice, the lactoferrin groups had cleared glucose as efficiently as the naïve group. Moreno-Navarrete *et al*. demonstrated that circulating lactoferrin was significantly decreased in patients with altered glucose tolerance and was negatively related to inflammatory markers [31]. In *ex vivo* experiments, a significant decrease in LPS-induced lactoferrin release from neutrophils was observed in subjects with type 2 diabetes [31]. Our study is the first observation in lactoferrin administration can improve insulin sensitivity as measured by OGTT and HOMA-IR.

HFSC stimulates overgrowth of intestinal microbiota, increasing intestinal permeability and leading to chronic inflammation [32]. In this study, HFCS (30%) induced overgrowth of fecal coliforms (data not shown) and higher serum LPS (Table 4). Our data indicate that lactoferrin altered the HFCS-induced imbalance in intestinal microbiota and reduced chronic inflammation. In previous studies, lactoferrin presents to exert anti-infectious and anti-inflammatory activities *in vivo*, and to inhibit LPS-induced IL-6 secretion in a human monocytic cell line [33]. This observation was extended with a demonstration that lactoferrin inhibits the expression of mRNA of proinflammatory cytokines, including TNF-α, IL-1, IL-6, and IL-8, and modulates the nuclear transcription factor kappa B (NF-κB) signaling cascade [34]. Lactoferrin has also been shown to downregulate IL-10 secretion in LPS-stimulated macrophages [35]. In our study, serum and hepatic LPS levels significantly increased in HFCS-induced HMMS, and this increase was significantly reduced IL-10 by lactoferrin (Table 4, 5). Significantly, reduced expression of TLR-4 was also observed in lactoferrin-treated groups indicated the reducing inflammatory cascade signaling (Figure 2). Puddu *et al*., indicate bovine lactoferrin conteracts TLR mediated activation signals in monocyte-derived dendritic cells [36]. Serum ALT (sALT) is a direct indicator of hepatitis, and lactoferrin reduced sALT in a dose-dependent manner. Furthermore, cytokine measurements indicated that lactoferrin could significantly reduce hepatic IL-1β, TNF-α, and MCP-1 (Table 5). Previous studies have shown that lactoferrin can bind LPS from *E. coli* and *Salmonella typhimurium* and remove the glycolipid from the bacterial surface [37]–[39]. Lactoferricin, a peptide produced by hydrolysis of lactoferrin by a gastrointestinal digestive enzyme, plays a central role in scavenging LPS [40]–[42]. Analysis of the structural characteristics of lactoferricin identified a six-residue sequence responsible for binding LPS of *E. coli* or *Pseudomonas aeruginosa* [43]–[45]. Serum and hepatic lactoferrin were measured by ELISA, and no significant differences were observed between the naïve and control groups (Table 4, 5). Interestingly, lactoferrin can be detected in serum and liver homogenates in a dose-dependent manner. This indicates that orally administered bovine lactoferrin can be absorbed into the circulation. Fischer *et al*. showed that orally administered lactoferrin could be detected in the liver, kidneys, gall bladder, spleen, and brain of the mouse within 10–20 min [46].

When IL-33 and TSLP, which are both epithelial-related pro-inflammatory cytokines, are present in tissue matrix, polarization in a type 2-environment allows precursor substances (IL-4 and IL-13) to activate dendritic cells to stimulate Th2 cells differentiation [47]. Our data indicate that lactoferrin can significantly reduce levels of the epithelial cell-derived cytokines IL-33 and TSLP (Table 4). Examination of pathological tissue sections also indicated that TSLP was significantly reduced in the lactoferrin-treated groups (Figure 2). On the other way, the adipocyte-derived plasma protein adiponectin has been shown to be decreased by obesity and to inhibit TNF-α-induced expression of endothelial adhesion molecules [48]. Adiponectin acts as an anti-inflammatory and anti-atherogenic plasma protein. Adiponectin is a biologically relevant endogenous modulator of vascular remodeling, linking obesity and vascular disease [49]. Adiponectin may downregulate IL-6 and TNF-α expression from Kupffer cells, thereby inhibiting inflammatory progression in the liver [50], [51]. Reducing visceral adipose tissue macrophages improves systemic glucose homeostasis, insulin sensitivity and increase adipnectin levels *via* CCR2/MCP-1 signaling in diet-induced obese mice [52], [53]. Our data suggest that upregulation of adiponectin expression by lactoferrin is correlated with anti-inflammatory status (Table 5) in modulation of tissue specific macrophage.

## Conclusions

In conclusion, treatment of mice with bovine lactoferrin had a twofold beneficial effect. Lactoferrin decreased the “first hit” by reducing serum triglyceride, cholesterol levels and retarding hepatic lipid accumulation. In addition, lactoferrin altered the “second hit” by and scavenging LPS in circulation to reduce the expression of hepatic inflammatory cytokines. As a natural substance, bovine lactoferrin contribute for the control of HFCS induced HMMS, including obesity, insulin resistance, hypertriglyceridemia, NAFLD and NASH.
